# Site-Directed Mutagenesis to Improve Sensitivity of a Synthetic Two-Component Signaling System

**DOI:** 10.1371/journal.pone.0147494

**Published:** 2016-01-22

**Authors:** Audrey Olshefsky, Laila Shehata, Natalie Kuldell

**Affiliations:** 1 Department of Biological Engineering, Massachusetts Institute of Technology, Cambridge, MA, United States of America; 2 BioBuilder Educational Foundation, Cambridge, MA, United States of America; University of Groningen, Groningen Institute for Biomolecular Sciences and Biotechnology, NETHERLANDS

## Abstract

Two-component signaling (2CS) systems enable bacterial cells to respond to changes in their local environment, often using a membrane-bound sensor protein and a cytoplasmic responder protein to regulate gene expression. Previous work has shown that *Escherichia coli’s* natural EnvZ/OmpR 2CS could be modified to construct a light-sensing bacterial photography system. The resulting bacterial photographs, or “*coli*roids,” rely on a phosphotransfer reaction between Cph8, a synthetic version of EnvZ that senses red light, and OmpR. Gene expression changes can be visualized through upregulation of a LacZ reporter gene by phosphorylated OmpR. Unfortunately, basal LacZ expression leads to a detectable reporter signal even when cells are grown in the light, diminishing the contrast of the *coli*roids. We performed site-directed mutagenesis near the phosphotransfer site of Cph8 to isolate mutants with potentially improved image contrast. Five mutants were examined, but only one of the mutants, T541S, increased the ratio of dark/light gene expression, as measured by β-galactosidase activity. The ratio changed from 2.57 fold in the starting strain to 5.59 in the T541S mutant. The ratio decreased in the four other mutant strains we examined. The phenotype observed in the T541S mutant strain may arise because the serine sidechain is chemically similar but physically smaller than the threonine sidechain. This may minimally change the protein’s local structure, but may be less sterically constrained when compared to threonine, resulting in a higher probability of a phosphotransfer event. Our initial success pairing synthetic biology and site-directed mutagenesis to optimize the bacterial photography system’s performance encourages us to imagine further improvements to the performance of this and other synthetic systems, especially those based on 2CS signaling.

## Introduction

As the field of synthetic biology grows, scientists and engineers are developing new tools to control living cells, thus expanding the range of input signals that can be used to control cellular gene expression. Recent advances include synthetic systems controlled by time [1], cellular density [2], and light [3]. These synthetic systems can be used to study a diverse collection of biological phenomena such as spatial dynamics, signal transmission patterns, and the effects seen from oscillatory input on signaling networks [4]. The bacterial photography system, developed by Levskaya et al in 2005 [5], is one early example of a light-sensitive synthetic system that successfully extends the toolkit for synthetic biology.

**Fig 1:**
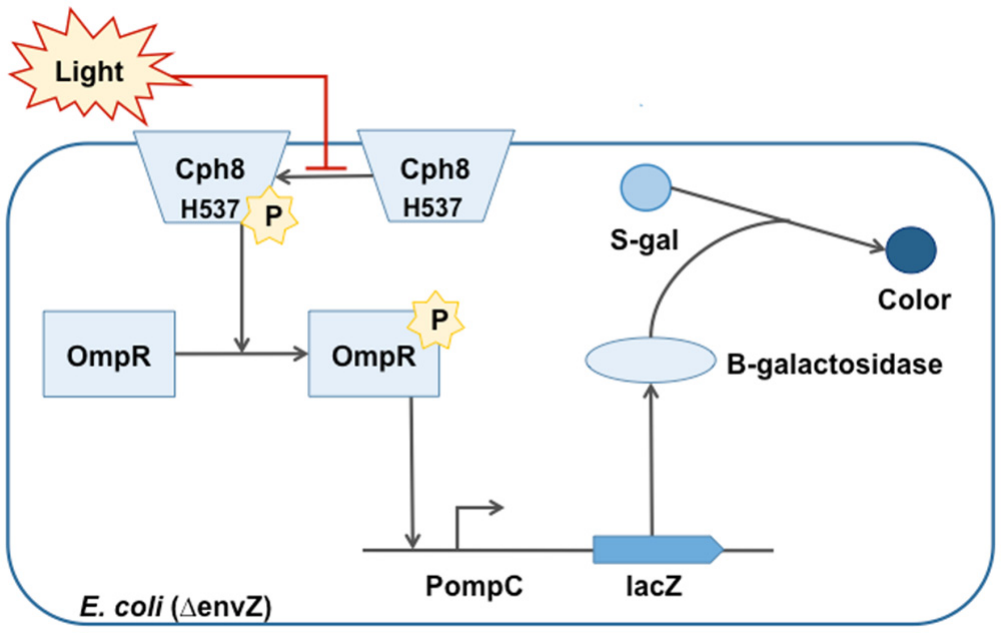
Phosphotransfers between the two-component signaling proteins in the bacterial photography system. The EnvZ-OmpR signaling system was modified for bacterial photography system with a light sensing protein introduced as a Cph1-EnvZ fusion protein called Cph8. The absence of light stimulates the autophosphorylation activity Cph8 protein. Cph8 transfers its phosphate to OmpR, increasing the affinity of OmpR for the PompC promoter and activating transcription of LacZ. The β-galactosidase expressed from the LacZ gene cleaves the S-gal in the media to leave a black precipitate. The mutagenized residue in Cph8, T541, influences the phosphotransfer reaction mediated by Cph8.

The bacterial photography system relies on a re-engineered two-component signaling system in *Escherichia coli* (*E*. *coli*). The cell’s natural EnvZ/OmpR signaling system was modified so cells could respond to light and deposit a black compound into the media when the modified cells were grown in the dark (Fig 1). When grown behind a black and white image printed onto a transparency, the cells develop the image into a bacterial photograph, or “*coli*roid,” based on which cells are grown behind the light portions of the image and which are grown in the dark. One modification to the EnvZ/OmpR signaling system that enables this novel cellular behavior is a light-sensing domain appended to EnvZ to generate a fusion protein called Cph8. The light-sensing domain is derived from a protein normally found in cyanobacteria, and the photoreceptor itself requires two phycocyanobillins as auxiliary factors for function. A second modification to the EnvZ/OmpR signaling system is the reporter gene, LacZ, that was introduced downstream of an OmpR-responsive transcriptional promoter. This reporter allows for visualization of the signaling activity in response to light. Finally, because the EnvZ/OmpR signaling system is normally used by *E*. *coli* to control the cell’s response to salt in the environment, the cell’s natural copy of the EnvZ gene was deleted from the genome.

Fully assembled, the bacterial photography system can display up to 10X differences in LacZ gene expression when comparing cells grown in the dark and the light, presumably through changes in the phosphorylation state and DNA binding properties of the engineered 2CS components. In the dark, the Cph8 light-sensor autophosphorylates at a histidine residue (H537) found on the EnvZ-portion of the protein. The sensor then acts as a kinase, transferring the phosphoryl group to an aspartic acid on OmpR. Phosphorylated OmpR has a higher affinity for the promoter, directing transcription of LacZ. Thus more β-galactosidase enzyme is produced in the dark, which can be visualized using one of several chromogenic compounds such as ONPG or S-gal. Cph8 can also act as a phosphatase, removing the phosphoryl group from the targeted aspartic acid on OmpR and resetting the system to express basal levels of LacZ when cells are exposed to red light. One common sequence motif found in many 2CS sensors is the HDLRT sequence that seems to regulate both the kinasing and the phosphatasing activity of the sensor. The first residue of this motif is H243 in EnvZ and H537 in Cph8.

In our lab, we measured up to three-fold differences in gene expression when the bacterial photography cells are grown in the dark versus the light, significantly less dramatic than the 10-fold difference described in the literature. Consequently, the color contrast in the dark versus light portions of the *coli*roids themselves was not as extreme as we would have liked. Though anecdotal evidence from colleagues suggests that this discrepancy with the published literature is likely to arise from a combination of experimental factors such as setting and procedural variations, we decided to use the subtle but detectable difference in dark versus light grown cells to explore genetic options for optimizing the signaling pathway. In particular, we mutagenized the final residue in the HDLRT motif, namely T541 in Cph8 which corresponds to T247 in EnvZ, to identify variants that might enhance the kinasing of OmpR, and thus make the dark portions of the *coli*roids even darker. This residue has been shown to affect signaling in the context of the natural EnvZ/OmpR signaling pair [6], and is less likely than other residues in the motif to disrupt EnvZ structure if mutated.

While some of the T541 mutants we identified did increase β*-galactosidase* activity in the dark, most mutants also affected the activity when cells were grown in the light, and so these mutants did not increase the contrast of the *col*iroid images. However, one mutant, namely the T541S mutant strain, did improve the contrast of the *coli*roid images, not by increasing expression of the reporter in the dark but rather by decreasing the reporter’s basal expression in the light. We suggest that this behavior arises from enhanced phosphatase activity by the chemically similar but physically smaller sidechain in Cph8 at position 541.

## Materials and Methods

### Bacterial Strains and Plasmids

The bacterial strain NB466 (genotype: MC4100 ara+ Φ(OmpC-lacZ) 10–25 ΔenvZ::KanR +pCph8 +pPL-PCBamp) (Levskaya 2005) was used to establish a baseline for β*-galactosidase* activity. The strain was constructed by transforming two plasmids involved in light sensing into kanamycin resistant *Escherichia coli* containing a fusion of an OmpC promoter with a LacZ reporter and lacking the histidine kinase, EnvZ. The chloramphenicol-resistant plasmid pCph8 contains a fusion protein of Cph1, a cyanobacterial photoreceptor, to EnvZ. The ampicillin resistant plasmid pPCB contains the ho1 and pcyA genes for the biosynthesis of phycocyanobilin, which allows for the photoreceptor to function. T541R, T541P, T541Y, T541L and T541S are mutant strains of pCph8 that were isolated in this study from a collection of Cph8 mutagenized at T541 as part of a laboratory class at MIT, 20.109. An H537A mutant was also constructed to serve as a negative control for Cph8 phosphorylation activity. Strain NB462 (genotype: MC4100 ara+ Φ(OmpC-lacZ) 10–25 ΔenvZ::KanR +pPL-PCBamp) (unpublished, Natalie Kuldell, MIT) was used for the transformation of the Cph8 mutants into bacteria. It lacks pCph8 but is otherwise genetically identical to strain NB466.

### Construction of Cph8 Mutants and Screen

A collection of Cph8 mutants was constructed and transformed into NB462 to screen for enhanced β*-galactosidase* activity when cells were grown in the dark. A modification of the Stratagene QuikChange Protocol was used to incorporate a degenerate oligonucleotide with “NNY” mutagenesis (where N represents any nucleotide and Y represents the pyrimidines) at the T541 site of Cph8 (QuikChange Site-Directed Mutagenesis Kit, Agilent Technologies, Santa Clara, CA). Oligonucleotides were incorporated into the pCph8 plasmid through a polymerase chain reaction.

The pool of mutagenized plasmids was transformed into NB462 via electroporation. DNA (4 μg) was added to 50 μL of electrocompetent NB462 cells and then incubated on ice for 1 minute. The cells were electroporated at 2.5 kV, followed by a 1000% V:V addition of SOC media (0.5% Yeast Extract, 2% Tryptone, 10 mM NaCl, 2.5 mM KCl, 10 mM MgCl2, 10 mM MgSO_4_, 20 mM Glucose) for cell recovery. Cells were incubated on a nutator at 37°C for 1 hour, then one-twenty-fifth and one-fifth of the cells were plated on MacConkey Lactose MUG Agar supplemented with ampicillin and chloramphenicol (50.1 g MacConkey Lactose MUG Agar per liter, 25 μg/mL ampicillin, 34 μg/mL chloramphenicol), on which higher β*-galactosidase* activity results in darker red colonies. The plates were incubated overnight at 37°C and several dark red colonies were selected and cultured overnight at 37°C for sequencing.

### Sequence Analysis

DNA was isolated from the candidate strains following the methods of Birnboim and Doly (Birnboim 1979). The bacterial cells were resuspended in 25 mM Tris pH 8, 10 mM EDTA pH 8 and 5 mM Glucose, then lysed in 1% SDS and 0.2 M NaOH. Non-plasmid DNA was precipitated in 3 M KAc pH 4.8, and the supernatants were microfuged with 100% ethanol to form pellets of plasmid DNA. The pellets were washed with 70% ethanol. After the remaining ethanol evaporated, the pellets were resuspended in 40 μL of sterile water. Plasmid DNA (800 ng) and sequencing primer N0296 (seq = TCG TCA ACC TCA TTT TGC GCC AG, 25 pmol) were combined with sterile water to a final volume of 15 uL, then sequenced by GENEWIZ (South Plainfield, NJ).

### β*-galactosidase* Assay

NB466 was cultured in liquid media in both the light and dark and assessed for β*-galactosidase* activity. Ampicillin, chloramphenicol and kanamycin were combined with 15 mL of Luria-Bertani broth at final concentrations of 25, 34 and 10 μg/mL respectively. Saturated overnight NB466 cultures were diluted into this broth at 1:1000. The inoculated media was divided evenly into two samples which were incubated overnight on a platform rocker at 37°C, one in the dark and one exposed to red filtered light (0.08–0.15 W/m2-650 nm range).

The enzymatic activity of the overnight liquid cultures was measured using a β*-galactosidase* assay. The OD600 was measured for dilutions of each overnight culture. A 100 μL aliquot of cells were then combined with Zbuffer (0.06 M Na2HPO4, 0.04 M NaH2PO4*H2O, 0.001 M KCl, 0.0001 M MgSO4) for a 1:4 V:V ratio. Cells were lysed by the addition of 0.1% SDS (20 μL) and chloroform (30 μL) and vortexed for 10 seconds. Ortho-nitrophenyl-β-galactoside to a final concentration of 0.8 mg/mL was added to each sample to begin the timed reactions. Reactions were stopped with the addition of Na_2_CO_3_ to a final concentration of 0.3 mM once the solutions were a light yellow color. All samples were microfuged for 1 minute at 13,000 RPM to separate out the chloroform, and the Abs420 and Abs550 were measured from the resulting supernatants. Negative controls lacking NB466 culture were prepared alongside these samples. β*-galactosidase* activity was calculated in Miller Units using the following equation, in which t is the reaction time in minutes and V is the volume of bacterial culture in milliliters in each reaction:

$$\text{1 Miller Unit = }1000\times \frac{\left(Abs420-1.75\times Abs550\right)}{\left(V\times t\times OD600\right)}$$

### Bacterial Photography

NB466 was cultured in the light and dark in solid media that contained an indicator of β*-galactosidase* activity. Ampicillin, chloramphenicol, and kanamycin were combined to final concentrations of 25, 34, and 10 μg/mL, respectively, with molten Luria-Bertani (15 mL) supplemented with 3,4-cyclohexenoesculetin-β-D-galactopyranoside (S-gal) (1% Tryptone, 0.5% Yeast Extract, 1% NaCl, 0.03% S-gal, 0.05% Ammonium Ferric Citrate, 1% Low Melting Point Agarose). To visualize enzymatic activity, saturated overnight cultures of NB466 were added for a 1:500 dilution, set in a 10 cm Petri dish for 30 minutes, and incubated at 37°C overnight, one in darkness and the other under red filtered light (0.08–0.15 W/m2 650 nm range).

A transparency of a black-and-white image was placed on the Petri dish and the culture was incubated overnight at 37°C under red filtered light (0.08–0.15 W/m2 650 nm range) to produce a *coli*roid.

### Western Analysis

The Cph8 protein expression of five mutant strains (T541R, T541Y, T541P, T541L, and T541S) was observed using a Western analysis. The OD600 of the wild type, NB462, kinase dead and mutant strains were measured and two ODs of each strain were microfuged for 1 minute. The resulting pellets were lysed with EasyLyse Bacterial Protein Extraction Solution (100 μL, Epicentre, Madison, WI), incubated at room temperature for 5 minutes, and microfuged for 2 minutes. Equal volumes of supernatant and SDS-PAGE Loading Dye (60 mM Tris pH 6.8, 2% SDS, 1% glycerol, 0.01% bromophenol blue, 5% beta-mercaptoethanol) were combined and each sample, along with a sample of H6-EnvZ protein, was boiled for 5 minutes. Samples were loaded onto a gel (Ready Gel 4–15% Tris H-Cl Gel, cat #161–1158, Bio-Rad Laboratories, Waltham, MA) in Running Buffer (25 mM Tris, 192 mM glycine, 0.1% SDS) alongside a “Kaleidoscope” protein molecular weight standard (Bio-Rad Laboratories, Waltham, MA) and the gel was powered at 200 V for 50 minutes. The gel was blotted to nitrocellulose using Transfer Buffer (25 mM Tris, 192 mM glycine, 20% v/v methanol) and the transfer was powered at 100 V for one hour. The resulting nitrocellulose filter was stored overnight in TBS-T Tris-Buffered Saline supplemented with Tween and milk (20 mM Tris pH 7.5, 500 mM NaCl, 0.1% Tween, 5% milk).

Anti-H6EnvZ antibody was diluted 1:1000 in TBS-T with milk and poured onto the nitrocellulose filter, then incubated on a platform shaker for 45 minutes. The filter was washed with TBS-T twice for five minutes on the platform shaker. Goat-antirabbit-alkaline phosphatase (Bio-Rad, Waltham, MA) was diluted 1:1000 in 15 mL TBS-T and added to the filter, then incubated for 30 minutes on the platform shaker. After two five-minute TBS-T washes, the blots were developed by incubating the filter on the platform shaker with alkaline phosphatase detection reagents (1 mL 25x detection stock + 25 mL H_2_0 with 0.25 mL solution A and 0.25 mL solution B, Alkaline Phosphatase Substrate Kit, Bio-Rad Laboratories, Waltham, MA).

The band intensities of the Western blot were measured using densitometric analysis on NIH ImageJ. A greyscale image of the blot was uploaded to the software, and gel analyzer options set to ‘Label with percentages’ and ‘Invert peaks’. The rectangular selection tool was used to draw a rectangle surrounding the first band, which was then measured using the gel analyzer tool. This process was repeated for the remaining bands, from which histograms were created using the ‘Plot Lanes’ tool. To subtract the background equally from all measurements, a line was drawn the same distance from the base of each plot, enclosing the area of the peak. The magic wand tool was clicked on each peak to measure its area, and the resulting measurements were normalized against those of the wild type strains.

## Results

We wanted to improve the bacterial photography system’s visual output by increasing the ratio of enzymatic activity between cells grown in the dark and the light. To achieve this, we performed site-directed mutagenesis at the codon for T541, which is near the phosphotransfer site of Cph8, in order to isolate mutants with increased β-galactosidase activity in the dark. Five of the mutants we isolated had distinct sequence changes in the region we mutagenized. We further examined these mutants using a Western blot to compare protein expression levels to those in the starting strain, and by assaying the mutant’s β-galactosidase activity after growth in the dark and the light. We also visually compared the *coli*roids produced by each of the strains.

### Characterization of the Initial Bacterial Photography System

**Fig 2:**
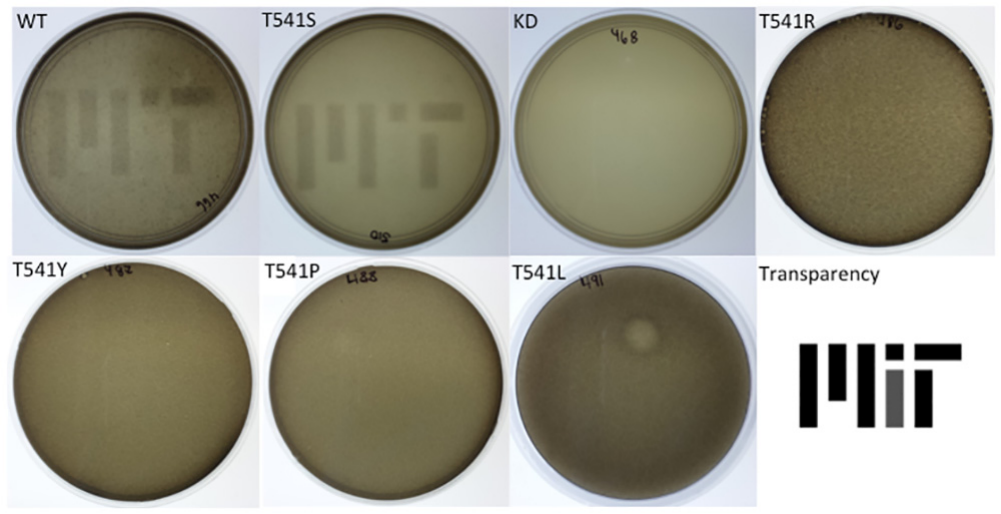
Bacterial photographs prepared with wild type and mutant strains reveal changes in visual contrast. Bacterial photographs were prepared in solid media for the strains indicated in the upper left corner of each panel using an identical image printed on transparency films. Incubation conditions consisted of red filtered light (0.08–0.15 W/m^2^, 650 nm range) at 37°C for 24 hours. WT: wild type, KD: kinase dead (H534A).

We began by characterizing the performance of the bacterial photography system that had been described by Levskaya et al (2005). Cells were grown in solid media supplemented with S-gal, which is cleaved by β-galactosidase to produce a black pigment. A transparency with the MIT logo was taped to the petri dish so that the red light in our incubator had to pass through the transparency to strike the cells. In this way, some of the cells growing in the supplemented media were growing in the light and other portions in the dark. After ~24 hours of incubation, a *coli*roid of the MIT logo was visible without the transparency because cells had reacted with the S-gal in the media to deposit black pigment depending on whether they were growing behind the light or dark portions of the image (Fig 2). Although the logo is clearly apparent, we believed greater contrast might be generated in the *coli*roid if the regions shielded from light had produced more precipitate or if the regions exposed to red light had produced less precipitate.

**Fig 3:**
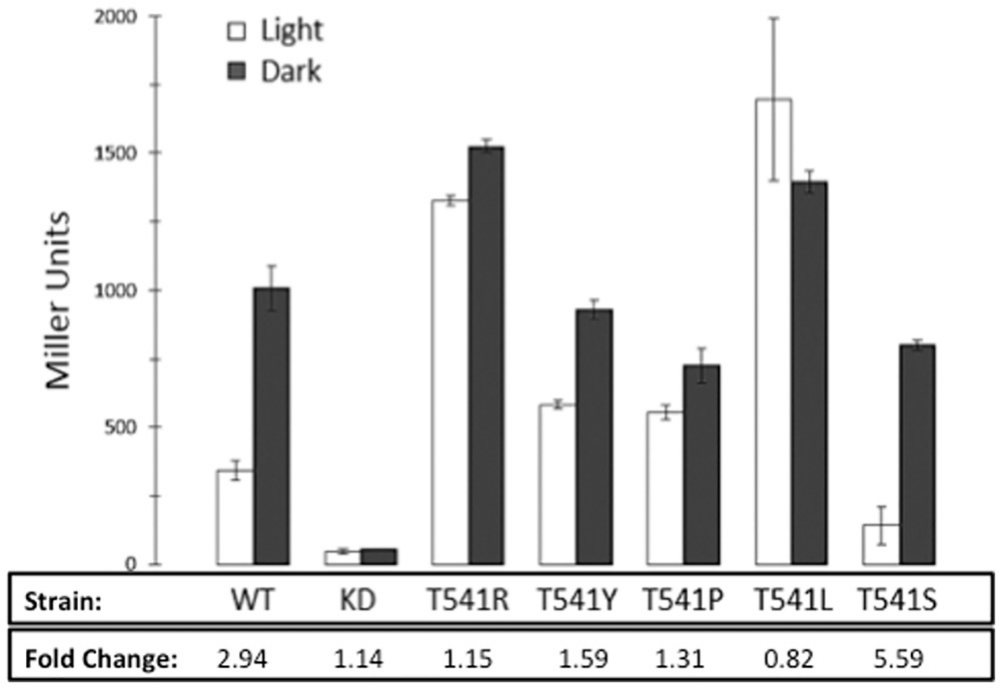
Comparison of light and dark β-galactosidase activity between wild type, kinase dead and mutant strains. Miller assays were performed in duplicate to measure β-galactosidase activity in Miller Units, as described in the Materials and Methods. The difference in β-galactosidase activity in the dark versus light (fold change) was calculated by dividing activity in the dark by activity in the light of the wild-type, kinase dead and mutant strains. Standard deviations are shown.

We used a β-galactosidase assay to quantify the enzymatic activity associated with liquid cultures of the bacterial photography system grown in the light and dark. The Miller Assay uses o-nitrophenyl-β-galactosidase (ONPG) as a substrate for β-galactosidase, thereby producing a yellow compound in proportion to the amount of enzyme present and the amount of time elapsed during the assay reactions. Cells that had been grown in the dark had an average of 1006 Miller Units of β-galactosidase activity, compared to the 342 Miller Units of activity in cells exposed overnight to red light (Fig 3). The approximate three-fold increase in enzyme activity when the cells were shielded from red light correlates with the differences observed in the solid media “*coli*roids” but was not as great as the 10-fold difference that had been reported previously.

### Mutagenesis of Cph8 at residue T541

**Fig 4:**
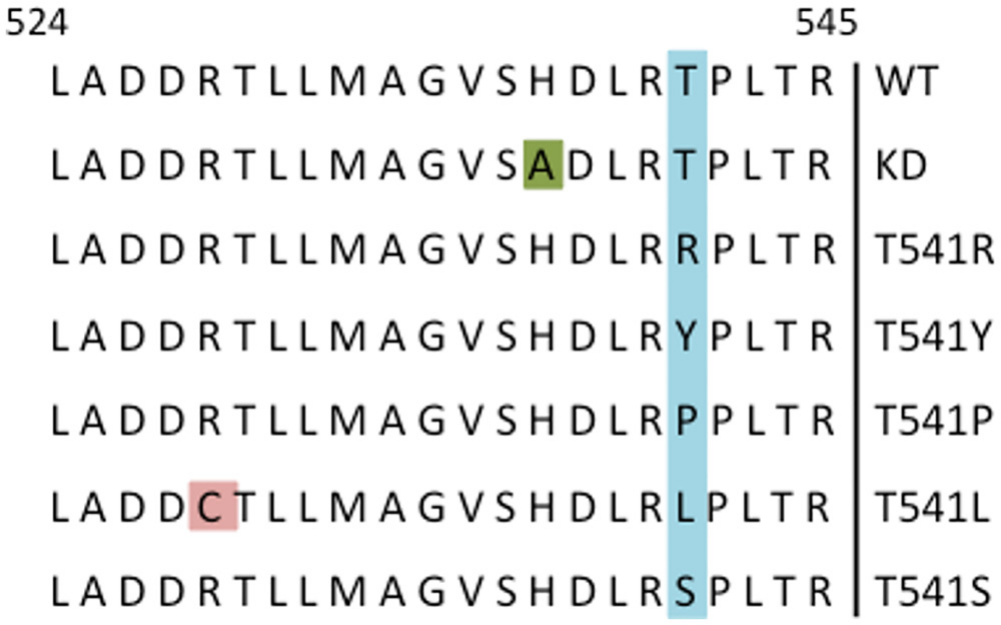
Amino acid sequences of mutants selected from Cph8 mutagenized screen. DNA sequencing was performed on DNA from the wild type strain (WT), the kinase dead strain (H537A), and five colonies selected from the Cph8 mutant hunt. The residue targeted by site-directed mutagenesis, T541, is highlighted in blue, and the H537A mutation expressed by the kinase dead strain is highlighted in green. An additional point mutation found at position 528 in strain T541L is highlighted in red. DNA sequences were not compared outside of the region encoding residues 524–545.

Believing a greater than three-fold difference in enzymatic activity was possible and that an increased ratio of dark to light activity would result in *coli*roids with greater contrast, we sought Cph8 mutants that would change the enzymatic output of the bacterial photography system. In particular, we sought mutations that might enhance the amount of phosphorylated OmpR when cells are growing in the dark, so called K^+^P^-^, because LacZ expression is upregulated when OmpR is phosphorylated. In this way, Cph8 mutations could lead to greater β-galactosidase activity in the portions of the media that were shielded from the red light. We chose to mutagenize Cph8 at the codon encoding T541, a position in the HDLRT region that had been identified by Hsing (1998) as critical for the OmpR kinase-dominant state (K^+^P^-^) of EnvZ. We used a mutagenic primer to vary the codon encoding T541, electroporated the mutagenized plasmids into cells carrying all but the Cph8 portion of the bacterial photography system, and grew the candidates on MacConkey Lactose indicator media. This electroporation and sequencing was carried out in the context of a laboratory class for undergraduates majoring in Biological Engineering at MIT [7]. Darker red colonies on MacConkey indicator media were selected for sequencing and further study because this phenotype could arise from elevated β-galactosidase activity. Five unique mutant strains were identified resulting in mutation of the amino acid at position 541 in Cph8 to arginine, tyrosine, proline, leucine and serine (Fig 4). The leucine mutant had an unexpected second mutation, C528A, that changed a residue 5’ to the HDLRT region previously identified as critical for regulating OmpR phosphorylation. DNA sequencing was performed on the entire Cph8 protein from the wild type and T541S strains (S1 Fig). No mutations aside from the T541S mutation were detected.

### Examination of T541 Mutants by Western Analysis

**Fig 5:**
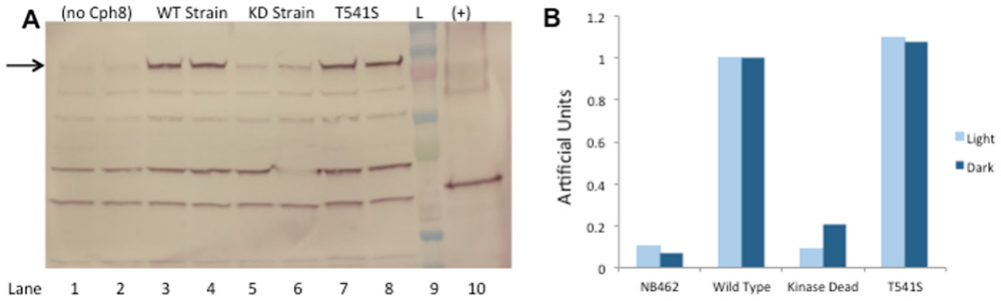
Western blot and densitometric analysis of Cph8 levels in T541S strain. Samples of the T541S strain, wild type strain, kinase dead strain and NB462, a strain lacking pCph8, were grown in light (odd numbered lanes) or dark (even numbered lanes) and compared with Western analysis. The expected band size of Cph8 is 83 kDa, indicated with a black arrow. (A) Lanes 1–2 are NB462, serving as a negative control for Cph8 protein. Lanes 3–4 contains wild-type Cph8, lanes 5–6 contains kinase dead and lane 7–8 is T541S mutant. Lane 9 (L) contains a Kaleidoscope ladder (Bio-Rad, Hercules, CA, USA). Lane 10 (+) is a truncated EnvZ protein tagged with His6, which serves as a positive control. (B) ImageJ densitometric analysis was used to calculate the relative darkness of sample bands through normalization to the optical intensity of the WT strains. This analysis resulted in quantitative approximations of the relative amounts of Cph8 protein produced per cell for each strain, as shown in the table.

Western analysis was performed on the wild type and mutant strains, comparing cells grown overnight in the light and the dark to understand if the variations in enzyme activity we detected for the Cph8 mutants could arise from variations in Cph8 protein concentration. As shown in Fig 5, the T541S mutant showed only slight differences in protein concentrations relative to the starting strain. By contrast protein concentrations were dramatically diminished for the kinase dead Cph8 mutation (H537A) as well as in the strain deleted for the Cph8 protein, which served as a negative control for the Western blot (Fig 5). Western analysis was performed on the other T541 mutant strains as well, with similar protein concentrations to that of T541S (data not shown).

### Examination of Enzymatic Activity in T541 Mutants

To quantify the β-galactosidase activity in the mutant strains, each was grown in liquid media either in the light and or in the dark, and Miller Assays were performed (Fig 3). Results were compared to the activity of the starting bacterial photography strain with Cph8 bearing the original T541 residue. Results were also collected for a mutant strain unable to undergo phosphotransferase reactions, namely a “kinase dead” mutant that bore the H537A mutation in Cph8. Compared to the approximate three-fold ratio of dark to light β-galactosidase activity in the original bacterial photography strain, several of the mutant strains unexpectedly showed more equivalent activity ratios. For example the T541R mutant was determined to express 983 and 519 Miller Units in the dark and the light, respectively. Only T541S improved the contrast in β-galactosidase activity between dark and light, changing the 3-fold difference seen in the starting strain to a 5.6-fold increase in the T541S strain.

To further characterize the mutants, *coli*roids were produced with each strain, growing the cells behind identical copies of the MIT logo mask. Consistent with the Miller Assay data, the mutants with diminished dark:light activity ratios failed to produce high contrast images. Indeed these mutants generally produced no *coli*roid image whatsoever. Only the starting strain and the T541S mutant produced *coli*roids (Fig 2), with a modest but noticeable improvement in the dark:light contrast generated with the T541S mutant strain.

## Discussion

Our study aimed to optimize a synthetic living system, namely the bacterial photography system, through site-directed mutagenesis and genetic screening. Five mutant strains were identified with altered β-galactosidase activity compared to the wild type strain. One of these mutants, T541S, appeared to increase the difference between the bacterial photography system’s output, not by increasing gene expression in the dark but rather by decreasing the reporter’s basal expression in the light. *Coli*roids prepared with the T541S mutant strain showed modestly improved visual contrast that could not be explained by differences in Cph8 protein expression, which appeared essentially identical by Western blot to Cph8 concentrations in the starting strain. An explanation for the T541S phenotype can be derived from the extensive mechanistic and structural studies of EnvZ. T247 in natural EnvZ, which corresponds to T541 in Cph8, has been shown to play a critical role in the active site of the His-Arg phosphotransfer scheme of EnvZ [8]. In particular, the hydroxyl group side-chain on the natural threonine seems to play a large role in the chemistry of the active site, functioning as an acid-base catalyst. Therefore, the T541S Cph8 mutant may have enhanced activity because it retains the hydroxyl group that is chemically similar to threonine at that position. This substitution allows the sidechain to satisfy both the helix formation requirements and the chemistry of the phosphotransfer scheme.

Unlike the T541S mutant, however, the other amino acid substitutions we examined diminished the light-sensitivity of the bacterial photography system. Four other mutant strains exhibited decreased ratios of β-galactosidase activity when cells were grown in the dark versus the light, and *coli*roids prepared with these strains showed a complete lack of contrast. It is possible that substitution of bulkier amino acids for the threonine affected conformational changes or access to the phosphotranferase active site. The limited light sensitivity of the mutant strains cannot be explained by a lack of Cph8 expression, however, as the Western blots for these strains show essentially wild-type levels of Cph8 protein. Moving forward, it would be interesting to determine if further mutation of the fusion protein could cause a faster and or more efficient response to red light. The screening method used in any additional studies should be improved, however, perhaps by an additional screening step to determine which colonies with improved β-galactosidase activity in the dark also have minimal β-galactosidase activity in red light. Combining such a two-step screen with the mutagenesis of new regions of Cph8 could uncover additional 2CS mechanisms of action. These discoveries could then speed the development or improvement of novel synthetic systems based on 2CS, such as the “ecolitaster” EnvZ-based biosensor that has been constructed to monitor vanillin concentration [9] or the EnvZ-based pathway that was designed to regulate the turbidity of cells in liquid culture [10]. Through this preliminary study, however, we have combined synthetic biology with more traditional molecular genetics and shown that it is possible to genetically optimize the performance of an engineered 2CS.

## Supporting Information

S1 Fig DNA sequences of the Cph8 protein from the wild type and T541S strains. DNA sequencing was performed on DNA from the wild type strain (WT) and the T541S mutant strain. The residue targeted by mutagenesis, T541, is noted in red. The HDLRT sequence, the region thought to regulate the kinasing and phosophatasing activity of the 2CS sensor, is bolded.
(TIF)

## References

[1] Danino T, Mondragón-Palomino O, Tsimring L, Hasty J. A synchronized quorum of genetic clocks. Nature. 2010; 463:326–30. doi: 10.1038/nature08753 20090747 10.1038/nature08753 PMC2838179

[2] Chu T, Huang Y, Hou M, Wang Q, Xiao J, Liu Q, et al *In Vivo* Programmed Gene Expression Based on Artificial Quorum Networks. Appl Environ Microbol. 2015; 81(17). 10.1128/AEM.01113-15 PMC4495192 25979894

[3] Gardner L, Deiters A. Light-Controlled Synthetic Gene Circuits. Curr Opin Microbiol. 2012; 16(3–4):292–299. 10.1016/j.cbpa.2012.04.010 PMC3424386 22633822

[4] Tischer D, Weiner OD. Illuminating cell signaling with optogenetic tools. Nat Rev Mol Cell Bio July 2014; 15:551–558. 10.1038/nrm3837 PMC4145075 25027655

[5] Levskaya A, Chevalier AA, Tabor JJ, Simpson ZB, Lavery LA, Levy M, et al Synthetic biology: Engineering *Escherichia coli* to see light. Nature 2005; 438:441–442. 16306980 10.1038/nature04405

[6] Hsing W, Russo FD, Bernd KK, Silhavy TJ. Mutations that alter the kinase and phosphatase activities of the two-component sensor EnvZ. J Bacteriol. 1998; 180(17):4538–4546. 9721293 10.1128/jb.180.17.4538-4546.1998 PMC107465

[7] Kuldell N, Hughes S, Stachowiak A. 20.109 Fall 2014 Lab Wiki: http://openwetware.org/wiki/20.109%28F14%29. Accessed on August 12, 2015.

[8] Dutta R, Yoshida T, Inouye M. The Critical Role of the Conserved Thr^247^ Residue in the Functioning of the Osmosensor EnvZ, a Histidine Kinase/Phosphotase, in *Escherichia coli*. J Biol. Chem. 2000; 275(38645–38653). 10.1074/jbc.M005872200 10973966

[9] Rodrigo G, Carreral J, Jaramillo A. ECOLITASTER: celular bioesnsor. *BMC Systems Biol*. 2007; 1(Suppl 1):1–2.

[10] You L, Cox RS, Weiss R, Arnold FH. Programmed population control by cell-cell communication and regulated killing. Nature. 2004; 428:868–871. 15064770 10.1038/nature02491

