# Supplementary figures and images for: Site-Directed Mutagenesis to Improve Sensitivity of a Synthetic Two-Component Signaling System
Source: PLoS One. 2016 Jan 22;11(1):e0147494. doi: 10.1371/journal.pone.0147494 (PMC4723039; doi:10.1371/journal.pone.0147494)

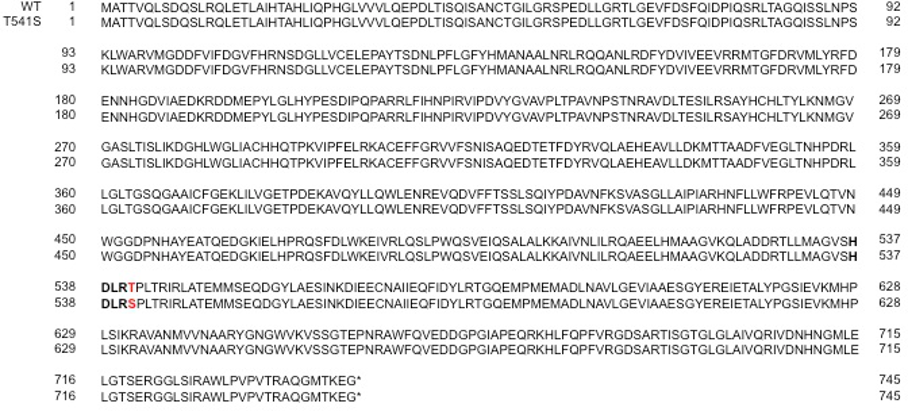

Supplement: S1 Fig — DNA sequencing was performed on DNA from the wild type strain (WT) and the T541S mutant strain. The residue targeted by mutagenesis, T541, is noted in red. The HDLRT sequence, the region thought to regulate the kinasing and phosophatasing activity of the 2CS sensor, is bolded. (TIF) [file pone.0147494.s001.tif]
